# Supplementary figures and images for: TV Interaction as a Non-Invasive Sensor for Monitoring Elderly Well-Being at Home
Source: Sensors (Basel). 2021 Oct 18;21(20):6897. doi: 10.3390/s21206897 (PMC8537784; doi:10.3390/s21206897)

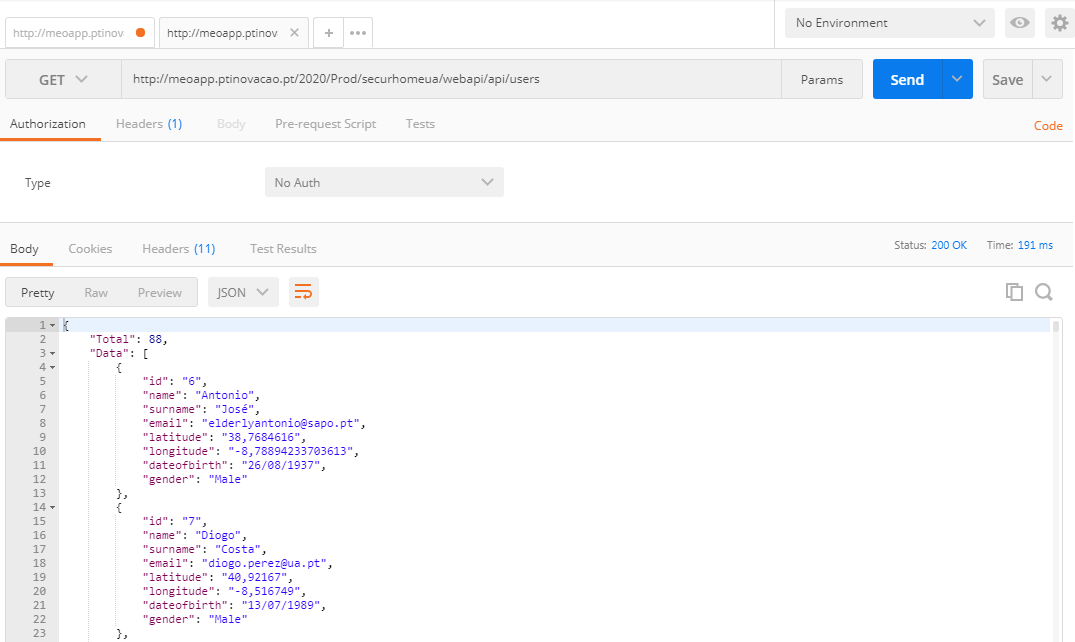

Supplement: Supplementary file 1 [file sensors-21-06897-s001.zip › Figure-S1.PNG]

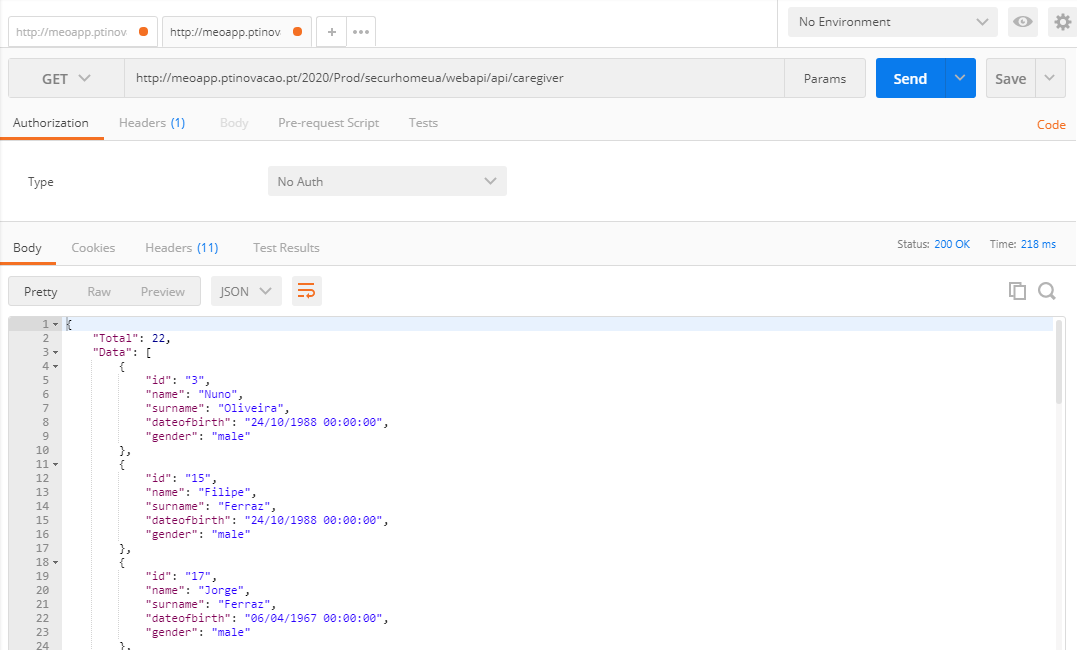

Supplement: Supplementary file 1 [file sensors-21-06897-s001.zip › Figure-S2.PNG]
